# A Case-Control Study of Maternal Periconceptual and Pregnancy Recreational Drug Use and Fetal Malformation Using Hair Analysis

**DOI:** 10.1371/journal.pone.0111038

**Published:** 2014-10-31

**Authors:** Anna L. David, Andrew Holloway, Louise Thomasson, Argyro Syngelaki, Kypros Nicolaides, Roshni R. Patel, Brian Sommerlad, Amie Wilson, William Martin, Lyn S. Chitty

**Affiliations:** 1 Institute for Women's Health, University College London, and University College London Hospitals NHS Foundation Trust, London, United Kingdom; 2 Harris Birthright Centre, King's College Hospital, London, United Kingdom; 3 Harris Birthright Centre, King's College Hospital, London, and Institute for Women's Health, University College London, United Kingdom; 4 Fetal Medicine Unit, St Michael's Hospital, Bristol, United Kingdom; 5 North-Thames Cleft Lip and Palate Unit, Great Ormond Street Hospital, London, United Kingdom; 6 Birmingham Women's Hospital, Birmingham, United Kingdom; 7 Clinical and Molecular Genetics Unit, UCL Institute of Child Health, and University College London Hospitals NHS Foundation Trust, London, United Kingdom; Hamamatsu University School of Medicine, Japan

## Abstract

**Objective:**

Maternal recreational drug use may be associated with the development of fetal malformations such as gastroschisis, brain and limb defects, the aetiology due to vascular disruption during organogenesis. Using forensic hair analysis we reported evidence of recreational drug use in 18% of women with a fetal gastroschisis. Here we investigate this association in a variety of fetal malformations using the same method.

**Methods:**

In a multi-centre study, women with normal pregnancies (controls) and those with fetal abnormalities (cases) gave informed consent for hair analysis for recreational drug metabolites using mass spectrometry. Hair samples cut at the root were tested in sections corresponding to 3 month time periods (pre and periconceptual period).

**Results:**

Women whose fetus had gastroschisis, compared to women with a normal control fetus, were younger (mean age 23.78±SD4.79 years, 18–37 vs 29.79±SD6 years, 18–42, p = 0.00001), were more likely to have evidence of recreational drug use (15, 25.4% vs 21, 13%, OR2.27, 95^th^CI 1.08–4.78, p = 0.028), and were less likely to report periconceptual folic acid use (31, 53.4% vs 124, 77.5%, OR0.33, 95^th^CI 0.18–0.63, p = 0.001). Age-matched normal control women were no less likely to test positive for recreational drugs than women whose fetus had gastroschisis. After accounting for all significant factors, only young maternal age remained significantly associated with gastroschisis. Women with a fetus affected by a non-neural tube central nervous system (CNS) anomaly were more likely to test positive for recreational drugs when compared to women whose fetus was normal (7, 35% vs 21, 13%, OR3.59, 95^th^ CI1.20–10.02, p = 0.01).

**Conclusions:**

We demonstrate a significant association between non neural tube CNS anomalies and recreational drug use in the periconceptual period, first or second trimesters, but we cannot confirm this association with gastroschisis. We confirm the association of gastroschisis with young maternal age.

## Introduction

Recreational drug use is reported to be associated with some fetal malformations, in particular, gastroschisis, brain and isolated limb defects. The Chief Medical Officer's annual report in 2004 highlighted a nearly three-fold rise in the prevalence of gastroschisis cases reported in the UK in the last 10 years [1]. Many studies have demonstrated a marked association between the incidence of gastroschisis and young maternal age [2], as well as the use of recreational drugs periconceptually and in pregnancy [3], [4]. These data come from epidemiological studies and questionnaire-based assessment of mothers and urine analysis, all of which are subject to poor ascertainment and unreliability [5]–[7]. In a small study using objective maternal hair root analysis for drug metabolites, we previously found evidence of periconceptual and/or first trimester maternal recreational drug use in 18% of cases with a diagnosis of gastroschisis compared with 0% in normal controls [8]. During this study we also detected evidence of recreational drug use in a few women with other fetal abnormalities, but the numbers were too small to permit any statistical analysis. It is important to expand and extend the study to examine the association with other congenital malformations that may be caused by vascular disruption, in order to objectively determine the role of recreational drugs in their aetiology.

There is evidence from animal studies that drugs such as metamphetamine and cocaine can cause a variety of fetal malformations including limb defects, brain anomalies, and abnormalities of the lip [9]–[12]. Observational studies have also indicated a possible association with fetal malformations in the human fetus including facial clefts, renal abnormalities, gastroschisis, and cardiac anomalies [13]–[16], and there are case reports of associated renal [17] and facial abnormalities [18]. An increased risk of gastroschisis and small bowel atresia has been associated with maternal use of vasoconstrictive drugs such as pseudoephedrine, phenylpropanol-amine, ephedrine, and methylenedioxymethamphetamine (MDMA) [19] as well as cocaine [20]. Vascular disruption has been proposed as a possible aetiology in the development of these abnormalities. The studies are small, however, and based on self-reporting of drug-taking with poor documentation of the timing of drug use, such that it is not possible to draw definitive conclusions.

The aim of this study was to determine whether maternal recreational drug use is a factor for the development of congenital fetal malformations that may be caused by vascular disruption such as gastroschisis, cleft lip and palate, renal, central nervous system and isolated limb defects. We hypothesized that women whose fetus has such a congenital malformation would be significantly more likely to have evidence of periconceptual, first or second trimester recreational drug use when compared to women whose fetus has an anomaly with no potential vascular aetiology or a normal fetus. Our previous study used mass spectrometry for the analysis of drug metabolites in maternal hair, the gold standard technique for forensic hair analysis that is highly sensitive and specific. This technique allows the temporal relationship between timing of drug exposure and embryogenesis to be explored, since evidence of drug use is ‘stored’ in the maternal hair for many months after ingestion, and the timing of drug exposure can be calculated from the length of the hair in which the drug is detectable [21], [22]. Thus drug use that predates embryogenesis and the development of fetal anomalies can be identified. Using similar methodology we studied pregnant women whose fetuses had a variety of congenital malformations, including those thought to be associated with vascular disruption. In particular we investigated the association with gastroschisis in a larger cohort of affected pregnancies.

## Methods

### Recruitment

This was a multicentre study (2006–2010). Women whose fetus had a congenital anomaly diagnosed on ultrasound scan were recruited as cases; those whose fetus was normal were recruited as controls. Cases were separated into two groups based on whether the anomaly had previously been associated with vascular disruption in preclinical and clinical studies [9]–[18] (see below).

### Ethics Statement

Informed written consent (Study number 05/Q0502/129, “Recreational drugs as aetiological factors in fetal malformations” approved by the Joint UCL/UCLH Committees on the Ethics of Human Research) was obtained from women attending the Fetal Medicine Units (FMUs) at University College London Hospital (UCLH), King's College Hospital (KCH), St Michael's Hospital Bristol (Bristol), Birmingham Women's Hospital (Birmingham), and the Cleft Lip and Palate Clinic at Great Ormond Street Hospital for Children, London (GOSH). Women whose fetus had a diagnosis of an abnormality were recruited sequentially from each Fetal Medicine Unit:

We recruited three groups of women:

1. *Vascular Disruption Cases*: those whose fetus had defects thought to be associated with vascular disruption (gastroschisis, isolated transverse limb defects, bowel atresia, facial cleft, cardiac and renal anomalies, and central nervous system anomaly (CNS) other than neural tube defect (NTD) “non-NTD CNS anomaly”).
2. *Non-Vascular Disruption Control Group*: those whose fetus had a defect not thought to have a vascular disruption aetiology and where there is no evidence of an association with recreational drug use (exomphalos, NTD, talipes and congenital diaphragmatic hernia (CDH), and aneuploidy, single gene disorders or genetic syndromes).
3. *Normal Control Group*: women whose fetus were normally-formed and had a normal karyotype.

Women in the normal control group were recruited at random in the antenatal clinics in the same hospitals and over the same time period after a normal detailed second trimester ultrasound scan had been performed. To take account of the younger age of women with pregnancies complicated by fetal gastroschisis, we ensured that each case of gastroschisis had a woman from the Normal Control Group of the same maternal age range (±2 years) recruited. In all cases and controls, ultrasound findings were confirmed after delivery by paediatric examination at birth, karyotyping and/or autopsy. There were no cases of women being recruited into the study on the basis of ultrasound findings that were not confirmed after delivery. Gestational age was confirmed by first trimester ultrasound in all women.

Information on women who declined to participate after being approached by the research team was collected prospectively. Women with hair shorter than 3 cm or those known to have hair extensions were not recruited. The Research Ethics Committee did not give permission to question the women about possible recreational drug use and self-reported drug use was not documented.

### Questionnaire

The primary aim of the study was to objectively investigate recreational drug use. However other social factors have previously been associated with fetal abnormalities and we therefore collected data from all women via a short questionnaire about these factors to enable adjustment as confounders in the statistical analysis: social history, smoking habits, alcohol use, use of over-the-counter and prescription drugs, including folic acid, paracetamol (acetaminophen) and non-steroidal anti-inflammatory drugs (NSAIDs) just prior to and during the current pregnancy. We used the Index of Multiple Deprivation (IMD) score, which combines a number of indicators chosen to cover a range of economic, social and housing issues, into a single deprivation score for individual neighbourhoods. The ID 2007 average IMD score was used (population weighted average of the combined IMD scores for the Super Output Areas in a district) and was derived from the woman's postcode according to National Statistics data. Information on their past obstetric history was collected from their antenatal booking history.

### Hair analysis method

Hair sampling and analysis was performed as previously described [8], after completion of the pregnancy, by Tricho-Tech Limited, Cardiff, UK. Once consent was obtained, a sample of hair was cut from the vertex (the crown) of the head as close to the scalp as possible. A sample of at least 50 hairs was obtained. The cut root ends were aligned and the sample was placed on aluminium foil with the cut root ends projecting about 1.5 cm from a marked end for clear identification. The foil was folded around the hair and securely kept in place. The sample was carefully packaged, barcoded for identification, sealed and sent to Tricho-Tech Limited for analysis of drug compounds which was performed blind to the clinical data pertaining to each case. The hair sample analysis was performed after washing the sample to remove any potential external contamination. The samples were then dried and cut into three 3-cm sections. Each section was weighed and the samples were analysed by enzyme-linked immunosorbent assay (ELISA) for the individual drug groups and any presumptive positives confirmed by gas chromatography with mass spectrometry (GCMS). GCMS is a powerful and widely used method of identifying and measuring analytes. Compounds become separated travelling through a GC column and those separated compounds enter the mass spectrometer, which creates charged particles (ions) from molecules. It then analyses those ions to provide information about chemical structure and molecular weight. Mass spectral patterns are reproducible and the mass spectra of known compounds are used to identify unknown substances.

Assays were performed for the following drugs or drug groups: amphetamine and its derivatives, such as methylenedioxy-methamphetamine (MDMA or ‘ecstasy’), barbiturates, benzodiazepines, cannabis and its metabolites, cocaine, ketamine, and opiates, including morphine, codeine, and dihydrocodeine. The limit of detection of the screening immunoassays was set at 10 ng/ml of hair extract. On a typical weight of sample of 10 mg this produces a limit of detection of 1 ng/mg of hair for the screening assays. The limit of detection of the GCMS confirmation assays was 0.2 ng/mg of hair. The GCMS confirmation assay for cocaine included measurement of cocaine and the major metabolite benzoylecgonine.

### Interpretation of hair results

**Figure 1:**
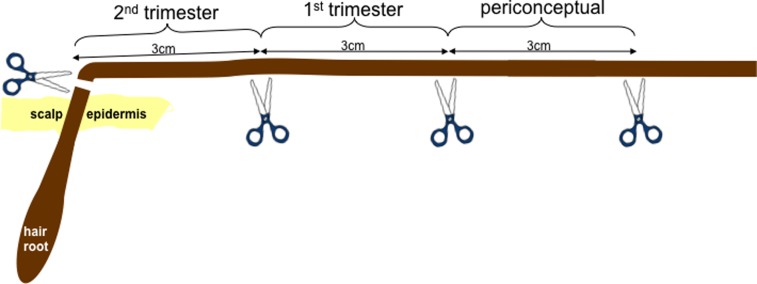
Hair samples comprising approximately 50 hairs were cut at the level of the hair root. From there they were divided into three 3 cm segments, representing the three time periods of interest: periconceptual (prior to the month in which conception took place and during the month in which conception took place), 1st trimester (up to and including 12 weeks of gestation) and 2nd trimester (13 weeks of gestation onwards). In this example the hair sample was cut when the woman was at the end of the 2nd trimester, giving a 3 cm length of hair for each time period of study.

The average rate of growth of hair is approximately 1 cm per month (range 0.75–1.5 cm); therefore, a 3-cm section of hair approximates to a three-month period [23]–[24]. Using this information and the gestational age at hair sampling, the timing of positive results was determined for each case and control and categorised into three groups periconceptual, 1^st^ trimester and 2^nd^ trimester (Figure 1). This method of analysis allows for the detection of drug use that occurred around the time that fetal organogenesis is being completed, when the development of a fetal structural anomaly may occur. The method of analysis, however, is not capable of reflecting exact drug doses taken, or the exact time point within the three-month period that drugs were used. For all analysed cases and controls, there was a hair sample available for the equivalent periconceptual and trimester time period, even for those women recruited in the late third trimester.

### Sample size calculation

Statistical advice was sought prior to study commencement. We based our sample size calculation on our primary hypothesis, that women whose fetuses had gastroschisis would be significantly more likely to have recreational drugs detected in their hair. In our previous study we detected drug use in 18% of the gastroschisis group [8]. Although no drug use was identified in the control group, we assumed that some women in the control group would be drug users. Between 2 and 3% of the world's population and 5.5% of mothers in the US are known to take recreational drugs [31]. Local figures (UCLH) suggested that 1–2% of booking women were users of recreational drugs. We therefore assumed that approximately 2% of the control group would be drug users.

Our sample size calculation was based on the Exact Sign Test: a sample size of 68 pairs on a 1∶1 basis or 46 gastroschisis Vascular Disruption Cases and 95 Normal Controls on a 1∶2 basis will have 90% power to detect a difference in proportions of 0.160 when the proportion of discordant pairs is expected to be 0.180. Women with pregnancies complicated by fetal gastroschisis are younger than the average pregnant population. This means that the control group for the gastroschisis cases would be on average younger than that required for the other abnormality groups. We therefore aimed to recruit 136 Normal Controls and 128 with ‘other’ abnormalities (Non-Vascular Disruption Control Group) to take account of this and to give sufficient flexibility to do an age-matched Normal Control group comparison for gastroschisis cases. For other specific fetal defects within the Vascular Disruption Group such as cleft lip and palate, congenital heart defects, brain and renal anomalies there is no objective data available on the level of drug use so we aimed to collect as many samples as possible during the time frame of the study.

### Statistics

To explore the relationship between the anomaly or group of anomalies and a possible associated factor, women with the Vascular Disruption type of fetal abnormality (renal, cardiac, CNS, etc) were compared with women in the recruited population whose fetus was normal (Normal Control group) and with those whose fetus had a Non-Vascular Disruption type of anomaly. Chi-squared tests (Pearson's test or Fisher's exact test for small numbers), Mann Whitney U or *t* tests were used to study the association between categorical or continuous variables. Binary logistic regression was then used to study factors that were independently found to be statistically significant, to explore confounding variables. The statistics package SPSS PASW Statistics version 21 was used for data analysis.

## Results

### Recruited women, exclusions and missing data

Eight women attending FMUs with a range of fetal abnormalities declined to participate in the study after being approached by the research team; no women with a normal fetus declined participation. Of 677 women who were recruited and from whom a hair sample was collected, three women were withdrawn due to young maternal age (<18 years), two women subsequently declined participation after hair collection, and 70 women were excluded due to collection of insufficient hairs (n = 58) or hair length too short (n = 12) for analysis. A further 85 women were excluded because fetal outcome could not be confirmed (n = 20), or the fetal anomaly did not fit the specified criteria (n = 65). For the remaining 517 women, hair was sent for analysis. Data was missing on ethnicity (25 cases, 4.8%), smoking and alcohol use (6 cases, 1.2%), over the counter medication use (4 cases, 0.8%) and prescription medication (3 cases, 0.6%, Table 1).

**Table 1:** Population demographics.

Category	Vascular Cases (n = 213)	Normal Controls (n = 161)	Non Vascular Controls (n = 143)	p	Total (%) n = 517
Maternal age in years, mean (median)	27.95 (28)	29.8 (30)	30.98 (30)	<0.00001	29.36 (29)
Maternal age range (years)	18–46	18–42	18–50	-	18–50
Ethnicity (no, %) Caucasian	151 (70.6)	125 (77.6)	103 (72.5)	0.131 (X^2^ 12.5, 8df)	379 (73.3)
Asian	29 (13.6)	23 (14.3)	17 (12.0)		69 (13.3)
Black	12 (5.6)	8 (5.0)	12 (9.2)		33 (6.4)
Mixed	4 (1.9)	3 (1.9)	4 (2.8)		11 (2.1)
Missing data	18 (8.4)	2 (1.2)	5 (3.5)		25 (4.8)
Primiparous (no, %)	119 (55.9)	89 (55.3)	76 (53.1)	0.875 (X^2^ 0.27, 2df)	284 (54·9)
Married or cohabiting (no, %)	168 (78.9)	135 (83.9)	112 (78.3)	0.385 (X^2^ 1.91, 2df)	415 (80.3)
Currently employed (no, %)	136 (63.8)	119 (73.9)	102 (71.3)	0.09 (X^2^ 4.83, 2df)	357 (69.1)
IMD score, mean (median)	26.61 (23.74)	26.89 (24.5)	25.10 (21.93)	0.523	26.27 (15.02)
IMD score range	2–83	2–59	1–68	-	1–83
**Smoking**: Current	32 (15.3)	21 (13.1)	18 (12.7)	0.74 (X^2^ 0.61, 2df)	71 (13.9)
In the last 6 months	28 (13.4)	15 (9.4)	11 (7.7)	0.16 (X^2^ 3.63, 2df)	54 (10.6)
Missing data	4 (1.9)	1 (0.6)	1 (0.6)	-	6 (1.2)
**Alcohol Use**: Current	34 (15.9)	32 (19.9)	12 (8.4)	0.018 (X^2^ 8.03, 2df)	78 (15.1)
In the last 6 months	81 (37.9)	77 (47.8)	46 (32.2)	0.019 (X^2^ 7.96, 2df)	204 (39.5)
Missing data	4 (1.9)	1 (0.6)	1 (0.6)	-	6 (1.2)
**Over the counter**: Folic acid	144 (67.3)	124 (77)	104 (72.7)	0.182 (X^2^ 3.40, 2df)	372 (72.0)
Paracetamol (acetaminophen)	36 (16.9)	38 (23.6)	26 (18.2)	0.258 (X^2^ 2.71, 2df)	100 (19.3)
NSAID use	11 (5.1)	11 (6.8)	7 (4.9)	0.721 (X^2^ 0.65, 2df)	29 (5.6)
Codeine/dihydrocodeine*	9 (4.2)	2 (1.2)	5 (3.5)	0.239 (X^2^ 2.86, 2df)	16 (3.1)
Missing data	2 (1.2)	1 (0.6)	1 (0.6)	-	4 (0.8)
**Prescription medication**: Antibiotics	29 (13.6)	22 (13.7)	26 (18.2)	0.735 (X^2^ 0.62, 2df)	73 (14.1)
Other prescription drugs	38 (17.8)	40 (24.8)	30 (21.0)	0.262 (X^2^ 2.89, 2df)	108 (20.9)
Missing data	1 (0.6)	1 (0.6)	1 (0.6)	-	3 (0.6)

NSAID: non-steroidal anti-inflammatory drug; df: degrees of freedom.

*Reported codeine/dihydrocodeine use.

### Demographic data

The mean maternal age was 29.36 years (SD 6.23 years). Further details of maternal social demographics including ethnicity are given in Table 1. Self-reported over-the-counter (105 women, 20.3%) or prescribed drug use (172 women, 33.3%) was common, with 13.7% of women currently smoking or drinking some alcohol (15.1%, Table 1). Mean gestational age at hair sampling was 24.18±6.45 weeks.

### Types of abnormalities

Table 2 shows the number and types of cases recruited by group. Women whose fetus had a minor anomaly such as mild ventriculomegaly, mild pyelectasis or echogenic foci were not recruited. In the Vascular Disruption Cases, the largest proportion recruited was women whose fetus had a gastroschisis (59 cases, 27.75% of Group). There were a variety of major cardiac, renal and Non-NTD CNS cases, and ten Other Vascular Cases that included transverse limb defects and bowel atresias. In the Non-Vascular Disruption Control Group, cases of exomphalos were the largest number (30 cases, 21% of Group), and the Genetic Cases were a variety of single gene disorders and fetuses with multiple anomalies but with normal karyotype.

**Table 2:** Number of women recruited per group.

Group	Number of women	% of total recruited	% of group
**Vascular Disruption Cases**			
Gastroschisis	59	11.4	27.7
Facial cleft	44	8.5	20.7
Renal anomaly	41	7.9	19.2
Cardiac defect	39	7.5	18.3
Non NTD Central Nervous System anomaly	20	3.9	9.4
*Other vascular	10	1.9	4.7
*Total Vascular Disruption Cases*	*213*	*41.2*	*100*
**Non-Vascular Disruption Control Group**			
Exomphalos	30	5.8	21.0
Congenital Diaphragmatic Hernia	29	5.6	20.3
NTD	25	4.8	17.5
Talipes	23	4.4	16.0
**Aneuploidy	21	4.1	14.7
ΔGenetic	15	2.9	10.5
*Total Non Vascular Disruption Controls*	*143*	*27.7*	*100*
**Normal Controls**			
*Normal structure and karyotype*	*161*	*31.1*	*-*
**Total Control Group (Non-Vascular Disruption and Normal)**	**304**	**58.8**	-
**Total recruited**	**517**	**100**	-

NTD: neural tube defect.

* Transverse limb defects (7 cases), bowel atresia (3 cases: duodenal atresia or ileal atresia) .

** Trisomy 21 (10 cases), trisomy 18 (2 cases), 1 case each of trisomy 13, 45 X0, XXX, triploidy, unbalanced translocation, 15q deletion, terminal deletion of chromosome 5.

Δ Skeletal dysplasia (8 cases), multiple abnormalities (6 cases), autosomal dominant polycystic kidney disease (2 cases).

Cardiac anomalies included large septal defects, hypoplastic left or right heart, Ebstein's anomaly, tetralogy of Fallot, transposition of the great arteries, left atrial isomerism, valvular atresias and double outlet right ventricle.

Other CNS anomalies included agenesis of corpus callosum, Dandy Walker spectrum, cerebellar hypoplasia, posterior fossa cyst, porencephalic cyst, severe ventriculomegaly and holoprosencephaly.

Renal anomaly: multicystic or dysplastic kidneys, lower urinary outflow tract obstruction.

### Recreational drug use

The presence of drugs was confirmed in the hair of 87 (16.8%) women. Ten women only had dihydrocodeine or codeine detectable (five periconceptually, four in the first trimester and one in the second trimester), of whom all reported this use as over the counter medication in the questionnaire. We therefore assumed that these drugs were taken for analgesic use only. After excluding them from analysis, there were 77 women (14.9%) with evidence of recreational drugs in their hair sample (Table 3). Ten women had evidence of poly-recreational drug use. The most common recreational drug was cannabis, detected in 68 women (13.2% of the study population). Cocaine was used by 18 women (3.5%); ten women (1.9%) used cocaine and cannabis together. In cocaine users, the majority (16, 88·9% of users) had the drug detectable in the second trimester hair sample, with eight women (44.4% of users) and five women (27.8% of users) with drug detectable in the first trimester and the periconceptual hair sample respectively, suggesting that drug use did not decrease during pregnancy. A minority of cannabis users (six women, 8.8% of users) only had the drug detectable in the periconceptual hair sample. Cannabis was detected in 39 women (55.7% of users) and in 52 women (76.5% of users) in the first and second trimester hair samples respectively. Amphetamines were detected in the hair of one woman, in combination with cocaine and cannabis. Ketamine was detected in the hair of one woman. No barbiturates or benzodiazepines were detected in any hair samples. Vasoactive drug use (cocaine or amphetamine) was detected in 18 women (22.2% of users).

**Table 3:** Presence of recreational drugs according to gestational time period analysed.

Drug type	Time period of recreational drug use			Recreational drug use at anytime	% of recreational drug users	% of total
	Periconceptual	1st trimester	2^nd^ trimester			
Cannabis	52	39	29	68	88·3	13.2
Cocaine	15	8	5	18	23·4	3.5
Ketamine	1	1	0	1	1·3	0.2
MDMA	1	0	0	1	1·3	0.2

MDMA: methylenedioxymethamphetamine.

### Recreational drug use and social factors

Women whose hair tested positive for recreational drugs had a higher median IMD deprivation score (33.34 compared to 22.04 in non-recreational drug users, p = 0·001, Table 4). Women testing positive for recreational drug use were younger than those testing negative (median maternal age 25.0 years compared to 29.5 years, p = 0·003). They were more likely to be smokers (current or within the last 6 months) and unemployed, compared to women who did not take recreational drugs (Table 4). Ethnicity and being single (no current partner) were not associated with recreational drug use. There was no association between use of alcohol, folic acid, aspirin, paracetamol (acetaminophen), antibiotics or other medication with recreational drug use.

**Table 4:** Social factors associated with recreational drug use.

Social factor	Recreational drug use			OR	95% CI	p value
	yes	no	total			
**Median IMD deprivation score**	33.34	22.04	-	—	-	0·001*
**Median maternal age (years)**	25.00	29.50	-	-	-	0·003*
**SmokingΔ (%)**	32 (26.0%)	91 (74.0%)	123 (100%)	2.75	1.65–4.58	0.000217**
**Non-smokers (%)**	44 (11.3%)	344 (88.7%)	388 (100%)			
**Unemployed (%)**	34 (21.3%)	126 (78.8%)	160 (100%)	1.97	1.20 to 3.23	0.011**
**Employed (%)**	43 (12.0%)	314 (88.0%)	357 (100%)			

Δsmoking currently or in the last 6 months; OR: Odds Ratio; CI: confidence interval; *Mann-Whitney U test; **Chi squared test.

### Recreational drugs and fetal anomaly

To explore the relationship between a specific anomaly and recreational drug use, women with that abnormality (renal, cardiac, CNS, etc) were compared with women in the Normal Control Group. Women with a fetus affected by a gastroschisis anomaly were significantly more likely to have recreational drugs detected in their hair when compared with women in the Normal Control Group (15, 25.4% vs 21, 13%, p = 0.028, Table 5). However when comparing an age-matched Normal Control group to women with a fetus affected by gastroschisis, there were no significant differences in detection of recreational drugs (15, 25.4% vs 12, 20.3%, p = 0.105).

**Table 5:** Type of fetal anomaly and recreational drug use.

Fetal anomaly	Recreational drug use (% of anomaly category)			OR	95%CI	p value
	yes	no	Total			vs Normal Control (X^2^, df)
**Normal Control Group**	21 (13%)	140 (87%)	161	-	-	-
**Vascular Disruption Cases**	36 (16.9%)	177 (83.1%)	213	-	-	-
Gastroschisis	15 (25.4%)	44 (74.6%)	59	2.27	1.08–4.78	0.028 (4.84, 1)
Non NTD CNS anomaly	7 (35.0%)	13 (65.0%)	20	3.59	1.29–10.02	0.010 (6.56, 1)
Facial cleft	4 (9.1%)	40 (90.9%)	44	0.67	0.22–2.05	0.48 (0.50, 1)
Renal anomaly	4 (9.8%)	37 (90.2%)	41	0.72	0.23–2.23	0.57 (0.33, 1)
Cardiac defect	3 (7.7%)	36 (92.3%)	39	0.56	0.16–1.97	0.36 (0.85, 1)
Other vascular	3 (30.0%)	7 (70.0%)	10	2.86	0.69–11.92	0.43 (1.70, 1)
**Non-Vascular Disruption Control Group**	20 (14%)	123 (86%)	143	-	-	-
Exomphalos	2 (6.7%)	28 (93.3%)	30	0.48	0.11–2.15	0.32 (0.97, 1)
Aneuploidy	1 (4.8%)	20 (95.2%)	21	0.33	0.04–2.62	0.27 (1.2, 1)
CDH	4 (13.8%)	25 (86.2%)	29	1.07	0.34–3.37	0.91 (0.012, 1)
Talipes	5 (21.7%)	18 (78.3%)	23	1.85	0.62–5.52	0.26 (1.25, 1)
NTD	6 (24.0%)	19 (76.0%)	25	2.12	0.75–5.87	0.15 (2.09, 1)
Genetic	2 (13.3%)	13 (86.7%)	15	1.03	0.22–4.87	0.98 (0.01, 1)

OR: Odds Ratio compared to Normal Control Group; CI: confidence interval; NTD: neural tube defect; df: degrees of freedom.

Women with a fetus affected by a non-NTD CNS anomaly were significantly more likely to have recreational drugs detected in their hair when compared with women in the Normal Control Group (7, 35% vs 21, 13%, p = 0.010, Table 5). There was no significant difference in maternal age between the groups (mean 29.85, SD 4.44, range 22–39 years vs mean 29.79, SD 5.597, range 18–42 years, p = 0.155).

Women with a fetus affected by a vascular disruption anomaly such as bowel atresia or transverse limb defect were more likely to test positive for recreational drug use, but this difference did not reach statistical significance. When the recreational drugs that are considered to be vasoactive (cocaine, MDMA, and amphetamines) were analysed separately from cannabinoids, the significant associations were lost, possibly due to the small numbers of positive cases. THC use alone was not significantly associated with any fetal anomaly.

There was no significant association between recreational drugs in the hair and the other anomaly groups: cardiac defect, renal, facial cleft, exomphalos, NTD, CDH, talipes, aneuploidy, and genetic (Table 5).

### Other factors analysed and fetal anomaly

The deprivation score (IMD) was significantly higher in women whose fetus was affected by talipes (mean IMD score 34.98± SD16.35) compared to women in the Normal Control Group (mean IMD score 26.89± SD13.82, p = 0.04), but IMD was not associated with any other fetal anomalies. Smoking, either current or past, was significantly more common in women with a fetus affected by a gastroschisis compared to the Normal Control Group (25 women, 43.1% vs 36 women, 22.5%, p = 0.04), but was not associated with any other fetal anomaly. When compared to the Normal Control Group, women with a fetus affected by a gastroschisis (19 women, 32.2% vs 26 women, 16.1%, p = 0.014) or a genetic syndrome (7 women, 46.7% vs 26 women, 16.1%, p = 0.009) were significantly more likely to be single, but this was not associated with any other fetal anomaly. There were no associations between alcohol use, ethnicity, or employment status with any fetal abnormality.

Compared to women in the Normal Control Group, women with a fetus affected by gastroschisis were significantly younger (Normal Control Group mean age 29.79± SD 6, range 18–42 vs gastroschisis mean age 23.78± SD 4.79, range 18–37 respectively, p = 0.00001), and those with a fetus affected by an isolated exomphalos or aneuploidy were significantly older compared to the Normal Control Group (exomphalos mean age 32.60± SD 7.03, range 18–50 p = 0.046; aneuploidy mean age 34.05± SD 7.40, range 18–45, p = 0.018). There was no association between maternal age and other fetal anomalies.

### Type of fetal anomaly and use of other drugs

Folic acid use was reported by 372 women (72%). There was no significant difference in the mean maternal age of folic acid users and non-users (29.63±5.82 versus 28.61±6.94 years respectively, p = 0.126). Women with a fetus affected by a gastroschisis were significantly less likely to report taking periconceptual folic acid (Table 6). There were no associations between folic acid use with any other fetal abnormality.

**Table 6:** Fetal anomalies and non-recreational drug use.

**Fetal anomaly**	**Periconceptual folic acid**					
	**yes**	**no**	**total**	**OR**	**95% CI**	**p value (X^2^, df)**
**Normal Control Group**	124 (77.5%)	36 (22.5%)	160	0.33	0.18–0.63	0.001 (11.99, 1)
**Gastroschisis**	31 (53.4%)	27 (46.6%)	58			
	**Prescription medication**					
	**Yes**	**no**	**total**	**OR**	**95% CI**	**p value (X^2^, df)**
**Normal Control Group**	60 (37.5%)	100 (62.5%)	160	-	-	-
**Gastroschisis**	12 (20.7%)	46 (79.3%)	58	0.43	0.21–0.89	0.020 (5.44, 1)
**Exomphalos**	5 (16.7%)	25 (83.3%)	30	0.33	0.12–0.92	0.035 (4.87, 1)

OR: Odds Ratio.

Missing cases were excluded.

Antibiotic prescription medication use was reported by 73 women (14.6%). Antiviral medication use, such as zanamivir or oseltamivir for treatment or prevention of influenza, was reported by six women (1.2%). Use of non-steroidal anti-inflammatory medication was reported by 29 women (5.6%). Prescription medication use other than antibiotics or antivirals was reported by 108 women (20.9%). There was no significant difference in the maternal age of prescription medication users and non-users. Women with a fetus affected by gastroschisis or exomphalos were less likely to report taking prescription medication (Table 6). There were no associations between the use of aspirin, paracetamol (acetaminophen), codeine, or DHC, and over-the-counter medication with any fetal abnormality.

### Regression analysis

There was more than one significantly associated factor in the analysis of women whose fetus had a gastroschisis. Binary logistic regression showed that after including all significantly associated variables, young maternal age still remained significantly associated with gastroschisis (Table 7) and no folic acid use nearly reached significance. Recreational drug use was not significantly associated with the presence of a gastroschisis.

**Table 7:** Binary logistic regression.

Variable	p value
Maternal age	*0.000002
Folic acid use	0.052
Prescription medication	0.090
Recreational drug use	0.398
Smoking currently or in the last 6 months	0.488
Married or living with partner	0.834

**Dependent variable is fetus with gastroschisis.**

There was no interaction between recreational drug use and maternal age in the analysis of women whose fetus had a non-NTD CNS anomaly. For exomphalos, binary logistic regression found that older maternal age and not taking prescription medication still remained significantly associated with exomphalos (Table 8).

**Table 8:** Binary logistic regression.

Variable	p value
Maternal age	*0.023
Prescription medication	*0.040

**Dependent variable is fetus with exomphalos.**

## Discussion

The main findings in this study are that using objective measurement in the hair, recreational drug use was higher in women whose fetus has a gastroschisis but this association no longer held when an age-matched control group was used or when other factors were considered. Only young maternal age remained significantly associated with fetal gastroschisis. In this cohort, as in our previous study [8] and many other studies, young women were significantly more likely to use recreational drugs, and young maternal age was strongly associated with gastroschisis. This current larger study has been able to tease out the confounding factors, the main one being young maternal age and shows that recreational drug use *per se* is not associated with gastroschisis.

In addition, we observed that recreational drug use was significantly higher in women whose fetus had a non-neural tube defect CNS anomaly. One previous study described ten infants with developmental delay and congenital cerebral anomalies who were found to have had *in utero* exposure to vasoactive drugs such cocaine and heroin [26], and another case has similarly reported a possible association of septo-optic dysplasia with multiple maternal recreational drug use [27]. Our study suggests a significant association between recreational drug use that includes vasoactive drugs and the non-vasoactive drug tetrahydrocannabinol, and CNS defects. Our findings should be validated further in a larger group of pregnancies where the fetus is affected by a non-neural tube defect CNS anomaly.

This is an objective study of recreational drug use in a large group of women with age-matched controls covering a broad range of fetal abnormalities. Our study shows that nearly 15% of women recruited had objective evidence of recreational drug use, mostly of cannabis, during the periconceptual and first trimester time periods, which reduced as pregnancy progressed. Recreational drug use was associated with higher deprivation score and younger maternal age.

A strength of the study is that we used an unbiased assessment of drug-taking, which did not rely on woman providing subjective information. The objective method used can assess recreational drug use over a period of weeks to months [6], [7], [22], [25], and avoids inaccurate, self, and usually under-reporting. This method allows for timing of drug exposure to be assessed by analysing hair at different distances from the hair root, as hair grows continuously at an average rate of 1 cm per month [8]. This methodology has been previously validated in forensic situations [8], [22]. Cosmetic treatments are known to alter drug concentration but do not eliminate drugs from the hair and studies demonstrate that the stability of drugs long term in the hair is high [7]. Other techniques, such as analysis of neonatal meconium, do not allow the timing of drug use to be elucidated. A weakness of the study is that it was not possible to measure the quantity of recreational drugs taken, nor the exact time period of the fetal exposure. In addition we relied on self-reporting of use of other drugs such as folic acid, NSAIDs, paracetamol (acetaminophen), over-the-counter and prescribed medication, and all are subject to recall bias. Finally, only a proportion of the recreational drugs identified in this study were in the vasoactive group of drugs that are considered to be associated with fetal defects. It is possible, therefore, that the inability to demonstrate a relationship between gastroschisis and recreational drug use in this study is due to the low numbers of amphetamine and cocaine users that we recruited. Nevertheless, our findings highlight the strong influence of maternal age and must call into question the previously held view that these vasoactive drugs are a cause of gastroschisis and its increasing prevalence.

Our findings differ to those published in a study by Draper et al [25] which demonstrated a significantly higher level of any recreational drugs and vasoconstrictive recreational drugs in particular, defined as cocaine, amphetamines, and ecstasy, in women with a fetal gastroschisis. This study differed from ours in a number of ways. In their group of 144 women with fetal gastroschisis and 432 matched normal controls, Draper et al performed hair analysis on only 39.5% of the cases and 26.3% of controls, and in the majority of women, the authors relied on self-reported recreational drug use and revised the estimates using data from hair analysis. When compared with our findings, the population in the study by Draper et al had a far lower prevalence of any type of recreational drug use and vasoactive recreational drug use in controls (5.5%) and cases (16.7%). In our study, vasoactive drugs were used by 22·2% of recreational drug users, but only 6.8% women with a fetal gastroschisis had evidence of this drug type in their hair. The different findings could also be due to the different populations studied, which in the Draper et al study were from three UK regional congenital anomaly registers, and the different methodologies used.

The incidence of recreational drug use in women whose fetus had anomalies that are considered to be vascular in origin, such as transverse limb defects and bowel atresias, was higher than in the rest of the cohort and although it did not reach statistical significance, this may reflect the small numbers of cases in this anomaly group. Unlike previous epidemiological studies [28], we did not find an association between facial clefts and recreational drug use.

Women whose fetus had a gastroschisis were significantly less likely to have taken folic acid periconceptually. This association however just failed to reach significance when other factors such as maternal age and prescription medication were taken into consideration. This may be due to the small numbers in our study. One observational case control study in Western Australia did not find any evidence of folate being an important factor in the prevention of birth defects other than neural tube defects [29]. It is not clear from the data, however, how many cases of gastroschisis were included in their analysis since the authors grouped “other” fetal abnormalities together for consideration (n = 119, intestinal atresias, choanal atresia, diaphragmatic hernia, gastroschisis, exomphalos, tracheo-oesophageal fistula). In another study, a Canadian birth defect registry, a significant increase in the prevalence of abdominal wall defects, and most notably gastroschisis, was observed after the introduction of folate fortification of grain products. The authors concluded that this was likely to be related to pre-existing increasing trends in gastroschisis incidence documented in several regions around the world rather than the effect of folic acid supplementation itself [30]. Draper et al [25] did not report periconceptual folic acid use in their study, but they had similar findings for maternal age, and smoking. Findings from a recent case control study supports the association of longer duration of folic acid use with a reduced risk of gastroschisis [32].

Our study shows that use of over-the-counter and prescription medication during pregnancy is common. Over 20% of women in this study reported use of prescription medication in their pregnancy, and nearly 15% reported antibiotic medication use. Use of non-steroidal anti-inflammatory medication was reported by 5.6% of women. The association of a reduced risk of gastroschisis in women who used prescription medication did not remain significant after adjusting for other factors such as maternal age, folic acid use, recreational drug use, smoking or having a partner. For exomphalos however, the association of a reduced risk remained after adjusting for maternal age, and the reason for this is unclear.

## Conclusion

In conclusion, this large study, using objective measurement of recreational drug exposure during the periconceptual period and the first and second trimesters of pregnancy, shows a high use of recreational drugs during pregnancy. Our findings strengthen the previously observed association between certain fetal anomalies, such as CNS defects and recreational drugs, but do not confirm the association with gastroschisis.

## References

[1] Chief Medical Officer's Annual Report (2004) Gastroschisis: a growing concern. London: Department of Health.

[2] Tan KH, Kilby MD, Whittle MJ, Beattie BR, Booth IW, et al (1996) Congenital anterior abdominal wall defects in England and Wales 1987–93: retrospective analysis of OPCS data. BMJ 313: 903–936. 8876090 10.1136/bmj.313.7062.903 PMC2352268

[3] Torfs CP, Velie EM, Oechsli FW, Bateson TF, Curry CJ (1994) A population-based study of gastroschisis: demographic, pregnancy and lifestyle factors. Teratology 50: 44–53. 7974254 10.1002/tera.1420500107

[4] Hume RF Jr, Gingras JL, Martin LS, Hertzberg BS, O'Connell K, et al (1994) Ultrasound diagnosis of fetal anomalies associated with in utero cocaine exposure: further support for cocaine-induced vascular disruption teratogenesis. Fet Diagn Ther 9: 239–245. 10.1159/000263940 7945904

[5] Grant T, Brown Z, Callahan C, Barr H, Streissguth AP (1994) Cocaine exposure during pregnancy: improving assessment with radioimmunoassay of maternal hair. Obstet Gynecol 83: 524–531. 8134061 10.1097/00006250-199404000-00007

[6] Kline J, Ng SK, Schittini M, Levin B, Susser M (1997) Cocaine use during pregnancy: sensitive detection by hair assay. Am J Pub Hlth 87: 352–358. 10.2105/ajph.87.3.352 PMC1381004 9096533

[7] Villain M, Cirimele V, Kintz P (2004) Hair analysis in toxicology. Clin Chem Lab Med 42: 1265–1272. 15576289 10.1515/CCLM.2004.247

[8] Morrison JJ, Chitty LS, Peebles D, Rodeck CH (2005) Recreational drugs and fetal gastroschisis: maternal hair analysis in the peri-conceptional period and during pregnancy. BJOG 112: 1022–1025. 16045512 10.1111/j.1471-0528.2005.00655.x

[9] Webster WS, Brown-Woodman PD (1990) Cocaine as a cause for congenital malformations of vascular origin: experimental evidence in the rat. Teratology 41: 689–697. 2353316 10.1002/tera.1420410605

[10] Yamamoto Y, Yamamoto K, Fukui Y, Kurishita A (1992) Teratogenic effects of metamphetamine in mice. Nippon Hoigaku Zasshi 46: 126–131. 1619809

[11] Colado ML, O'Shea E, Granados R, Misra A, Murray TK, et al (1997) A study of the neurotoxic effect of MDMA ('ecstasy') on 5-HT neurones in the brains of mothers and neonates following administration of the drug during pregnancy. Br J Pharmacol 121: 827–833. 9208155 10.1038/sj.bjp.0701201 PMC1564752

[12] He N, Lidow MS (2004) Cerebral cortical abnormalities seen in non-human primate model of prenatal cocaine exposure are not related to vasoconstriction. Neurotoxicology 25: 419–432. 15019305 10.1016/j.neuro.2003.10.002

[13] Plessinger MA (1998) Prenatal exposure to amphetamines. Risks and adverse outcomes in pregnancy. Obstet Gynecol Clin North Am 25: 119–38. 9547763 10.1016/s0889-8545(05)70361-2

[14] Nora JJ, Vargo TA, Nora AH, Love KE, McNamara DG (1970) Dexamphetamine: a possible environmental trigger in cardiovascular malformations. Lancet 1: 1290–1291. 10.1016/s0140-6736(70)91765-4 4192516

[15] Bays J (1994) Fetal vascular disruption with prenatal exposure to cocaine or metamphetamine. Pediatrics 87: 416–8. 2000289

[16] Lipshultz SE, Frassiac JJ, Orav EJ (1991) Cardiovascular abnormalities in infants prenatally exposed to cocaine. J Pediatr 118: 44–51. 1986097 10.1016/s0022-3476(05)81842-6

[17] Kashiwagi M, Chaoui R, Stallmach T, Hurlimann S, Lauper U, et al (2003) Fetal bilateral renal agenesis, phocomelia, and single umbilical artery associated with cocaine abuse in early pregnancy. Birth Defects Res A Clin Mol Teratol 67: 951–952. 14745934 10.1002/bdra.10101

[18] Markov D, Jacquemyn Y, Leroy Y (2003) Bilateral cleft lip and palate associated with increased nuchal translucency and maternal cocaine abuse at 14 weeks of gestation. Clin Exp Obstet Gynecol 30: 109–110. 12854855

[19] Werler MM, Sheehan JE, Mitchell AA (2003) Association of vasoconstrictive exposures with risks of gastroschisis and small intestinal atresia. Epidemiology 14: 349–354. 12859037

[20] Hume RF Jr, Martin LS, Bottoms SF, Hassan SS, Banker-Collins K, et al (1997) Vascular disruption birth defects and history of prenatal cocaine exposure: A case control study. Fet Diagn Ther 12: 292–295. 10.1159/000264488 9430211

[21] Klein J, Karaskov T, Koren G (2000) Clinical applications of hair testing for drugs of abuse - the Canadian experience. Forensic Sci Int 107: 281–288. 10689580 10.1016/s0379-0738(99)00171-1

[22] Spiehler V (2000) Hair analysis by immunological methods from the beginning to 2000. Forensic Sci Int 107: 249–259. 10689577 10.1016/s0379-0738(99)00168-1

[23] Nakahara Y, Shimamine M, Takahashi K (1992) Hair analysis for drugs of abuse. III. Movement and stability of metoxyphenamine (as a model compound of methamphetamine) along hair shaft with hair growth. J Analytic Toxicol 16: 253–257. 10.1093/jat/16.4.253 1501478

[24] Miyazawa N, Uematsu T (1992) Analysis of ofloxacin in hair as a measure of hair growth and as a time marker for hair analysis. Ther Drug Monit 14: 525–528. 1485377 10.1097/00007691-199212000-00015

[25] Draper ES, Rankin J, Tonks AM, Abrams KR, Field DJ, et al (2008) Recreational drug use: a major risk factor for gastroschisis? Am J Epidemiol 167: 485–491. 18063593 10.1093/aje/kwm335

[26] Dominguez R, Aguirre Vila-Coro A, Slopis JM, Bohan TP (1991) Brain and ocular abnormalities in infants with in utero exposure to cocaine and other street drugs. Am J Dis Child 145: 688–695. 1709777 10.1001/archpedi.1991.02160060106030

[27] Orrico A, Galli L, Zappella M, Monti L, Vatti GP, et al (2002) Septo-optic dysplasia with digital anomalies associated with maternal multidrug abuse during pregnancy. Eur J Neurol 9: 679–682. 12453085 10.1046/j.1468-1331.2002.00473.x

[28] Beaty TH, Wang H, Hetmanski JB, Fan YT, Zeiger JS, et al (2001) A case-control study of non-syndromic oral clefts in Maryland. Ann Epidemiol 11: 434–442. 11454503 10.1016/s1047-2797(01)00222-8

[29] Bower C, Miller M, Payne J, Serna P (2006) Folate intake and the primary prevention of non-neural birth defects. Aust NZ J Public Health 30: 258–261. 10.1111/j.1467-842x.2006.tb00867.x 16800203

[30] Godwin KA, Sibbald B, Bedard T, Kuzeljevic B, Lowry RB, et al (2008) Changes in frequencies of select congenital anomalies since the onset of folic acid fortification in a Canadian birth defect registry. Canadian J Pub Hlth 99: 71–75. 10.1007/BF03403753 PMC6975565 18767269

[31] Huestis MA, Choo RE (2002) Drug abuse's smallest victims: in utero drug exposure. For Sci Int 2002 128: 20. 10.1016/s0379-0738(02)00160-3 12208017

[32] Paranjothy S, Broughton H, Evans A, Huddart S, Drayton M, et al (2012) The role of maternal nutrition in the aetiology of gastroschisis: an incident case-control study. Int J Epidemio l41: 1141–1152. 10.1093/ije/dys092 22798661

